# The phosphatase activity of soluble epoxide hydrolase regulates vascular calcification through the metabolism of pyrophosphate anions

**DOI:** 10.1038/s41419-025-08390-6

**Published:** 2025-12-27

**Authors:** Hind Messaoudi, Olivier Varennes, Elodie Berg, Nicolas Perzo, Sylvanie Renet, Ghiles Chegrani, Thomas Duflot, Guillaume Feugray, Felix F. Lillich, Gilles Kauffenstein, Valéry Brunel, Isabelle Six, Romuald Mentaverri, Vincent Richard, Ignacio Anegon, Christophe Morisseau, Saïd Kamel, Ewgenij Proschak, Jérémy Bellien

**Affiliations:** 1 Univ Rouen Normandie, Inserm, EnVI UMR 1096, Rouen, France; 2 EA 7517, Mécanismes Physiopathologiques et conséquences des calcifications cardiovasculaires (MP3CV), Centre de Recherche Universitaire en Santé, Université de Picardie Jules Verne, Amiens, France; 3 Department of Pharmacology, CHU Rouen, Rouen, France; 4 Department of General Biochemistry, CHU Rouen, Rouen, France; 5 Institute of Pharmaceutical Chemistry, Goethe-University, Frankfurt am Main, Germany; 6 Inserm UMR 1260, CRBS, Strasbourg University, Strasbourg, France; 7 Nantes Université, CHU Nantes, Inserm, CNRS, SFR Santé, Inserm UMS 016, CNRS UMS 3556, Nantes, France; 8 Nantes Université, CHU Nantes, Inserm, Centre de Recherche en Transplantation et Immunologie, UMR 1064, ITUN, Transgenesis Rat ImmunoPhenomic Platform, Nantes, France; 9 Department of Entomology and Nematology, and UCD Comprehensive Cancer Center, University of California, Davis, CA USA

**Keywords:** Calcification, Enzyme mechanisms

## Abstract

While the hydrolase activity of soluble epoxide hydrolase (sEH) reduces vascular calcification, it is not known whether the phosphatase activity of sEH (sEH-P) is also involved. Pharmacological and genetic inhibition of sEH-P reduced the increased calcium deposition in rat aortic rings cultured under high-phosphate conditions. This was associated with decreased mRNA expression of the osteochondrogenic markers *Msx2* and *Sox9*. Deendothelialization of the aortic rings abolished this anticalcifying effect, while the calcification of human aortic smooth muscle cells was unaffected by sEH-P inhibition, suggesting a predominant role of the endothelium. Endothelial NO release did not appear to contribute, but an increased level of the calcification inhibitor pyrophosphate anions (PPi) was observed in the culture supernatant of aortic rings when sEH-P was inhibited. In vitro experiments demonstrated that PPi is a substrate of sEH-P, and that inhibiting sEH-P prevented the high-phosphate induced decrease of PPi in human aortic endothelial cells. Furthermore, the aortic calcification related to chronic kidney disease induced by subtotal nephrectomy was reduced in sEH-P-deficient rats compared to wild-type rats. This was associated with an improvement in flow-induced isolated mesenteric artery dilatation and a reduction of cardiac hypertrophy and fibrosis. Vascular calcification is regulated by sEH-P through the metabolism of endothelial PPi. The prevention of vascular calcification, together with the reduction in vascular dysfunction and cardiac remodeling, suggests that inhibiting sEH-P may help to prevent the cardiovascular complications associated with chronic kidney disease.

## Introduction

Vascular calcification is an abnormal deposition of calcium in the blood vessel wall, driven by an imbalance between inducers such as elevated inorganic phosphate and pro-calcific signaling pathways, and inhibitors such as pyrophosphate anions (PPi) that normally prevent mineral deposition [1–4]. When this balance shifts, key osteogenic and chondrogenic transcription factors such as Msh Homeobox 2 (Msx2), SRY-Box Transcription Factor 9 (Sox9), and Runt-Related Transcription Factor 2 (Runx2) are upregulated, promoting the transition of vascular smooth muscle cells (VSMCs) toward bone- and cartilage-like cell types [1–4]. At the same time, markers of the contractile smooth muscle phenotype, such as Smooth Muscle Myosin Heavy Chain (SMMHC), are often downregulated [1–4]. In addition, tissue-nonspecific alkaline phosphatase (TNAP) promotes the extracellular degradation of PPi, further removing a key barrier to hydroxyapatite crystal formation, which is the primary mineral component of the calcified matrix [1–4]. Medial calcification is commonly observed in patients with chronic kidney disease (CKD) and diabetes [1–4]. In these conditions, disturbances in mineral metabolism, including hyperphosphatemia and secondary hyperparathyroidism, promote a pro-calcific environment that drives VSMCs to adopt osteochondrogenic features. This process is compounded by inflammation, oxidative stress, and a reduction of endogenous inhibitors such as PPi [1–4]. The extent and severity of arterial calcification contribute significantly to increased arterial stiffness and elevated cardiovascular morbidity and mortality in these patient populations. Currently, no specific therapeutic strategies are available to prevent or treat the development of vascular calcification, highlighting an important therapeutic need.

In this context, soluble epoxide hydrolase (sEH) could be a promising new therapeutic target. The sEH is a bifunctional enzyme expressed especially in the cytosol of endothelial cells and VSMC [5, 6]. It hydrolyzes through its C-terminal domain (sEH-H) CYP450-derived epoxyfatty acids, particularly epoxyeicosatrienoic acids (EETs), which have potent anti-inflammatory and vasodilatory properties, into the corresponding diols, dihydroxyeicosatrenoic acids (DHETs) [5–7]. In addition, sEH possesses a less studied phosphatase activity (sEH-P) in its *N*-terminal domain. The sEH-P promotes the intracellular hydrolysis of molecules containing a pyrophosphate moiety and has been shown to metabolize various lipid mediators, including lysophosphatidic acids (LPA), into monoacylglycerols (MAG) [8]. Recently, we demonstrated that sEH-P contributes to cardiometabolic homeostasis through the regulation of LPA-mediated PPARγ activation [9]. Pharmacological inhibition of sEH-H has emerged as a new therapeutic strategy to prevent the cardiovascular complications associated with various diseases by restoring EETs bioavailability [5–7]. Nevertheless, endothelium-derived EETs have been shown to be pro-calcifying molecules at least in vitro and therefore, sEH-H may prevent calcification through their hydrolysis [10]. However, sEH knockout, which eliminates both sEH activities, was recently shown to prevent the development of aortic calcification including in vivo in a mouse model of chronic kidney disease [11]. In addition, the sEH Arg287Gln polymorphism, known to decrease sEH-H activity towards EETs but also to modulate sEH-P activity, was associated with the presence of coronary and carotid artery calcified plaques [12–14]. These results strongly support the hypothesis that sEH-P is also involved in the pathophysiology of vascular calcification.

In this context, this study aimed to assess the effects and the underlying mechanisms associated with the pharmacological and genetic inhibition of sEH-P in cell and tissue calcification assays in vitro and in an in vivo model of CKD-induced vascular calcification.

## Results

### sEH-P inhibition prevents the calcification of rat aortic rings

**Fig. 1:**
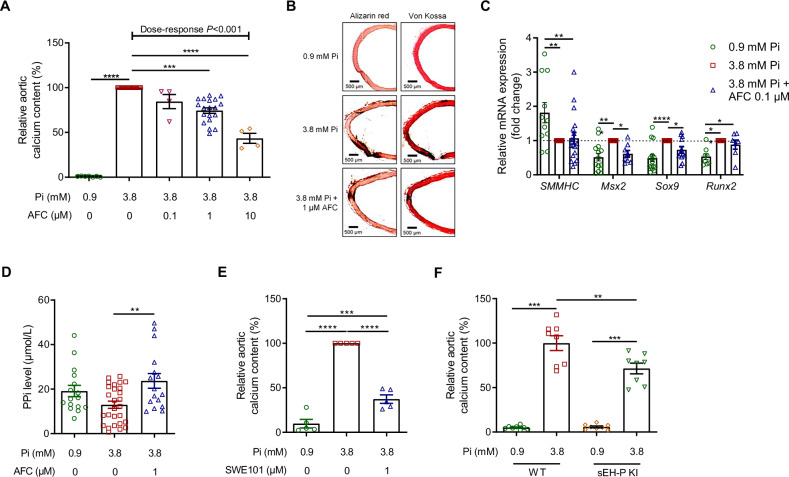
Inhibition of sEH-P prevents the ex vivo calcification of aortic rings. Relative calcium content of aortic rings cultured during 7 days under normal (0.9 mM inorganic phosphate, Pi) and high-phosphate (3.8 mM Pi) conditions, in absence and in presence of increasing concentrations of the sEH-P inhibitor *N*-acetyl-S-farnesyl-L-cysteine (AFC; **A**), and representative images of Alizarin red and Von Kossa staining (**B**). Aortic mRNA expression levels of the contractile marker smooth muscle myosin heavy chain (*SMMHC*) and of the osteochondrogenic markers Msh homeobox 2 (*Msx2*), sex determining region Y-box 9 (*Sox9*) and runt-related transcription factor 2 (*Runx2*) (**C**) and pyrophosphate anions (PPi) levels in culture supernatants (**D**) after 7 days of culture under 0.9 and 3.8 mM Pi conditions, in absence and in presence of AFC 1 µM. Relative aortic calcium content of aortic rings cultured during 7 days in 0.9 and 3.8 mM Pi in absence and in presence of the sEH-P inhibitor SWE101 (**E**) and of aortic rings isolated from WT and sEH-P KI rats cultured during 7 days in 0.9 and 3.8 mM Pi (**F**). Mean and SEM values are shown and group effect was determined by one-way *ANOVA* with Bonferroni’s *post hoc* test. *P < 0.05, **P < 0.01, ***P < 0.001, *** *P* < 0.0001.

Inhibition of sEH-P, using *N*-acetyl-S-farnesyl-L-cysteine (AFC) [15], induced a dose-dependent decrease in the calcium content of aortic rings cultured under high-phosphate conditions (Fig. 1A). These results were illustrated by Alizarin red and Von Kossa staining, which showed increased calcium deposits in aortic rings (Fig. 1B). Of note, this effect was obtained with a concentration of AFC of 1 µM that did not reduce aortic viability in contrast to 10 µM (Supplementary Fig. 1). AFC at 1 µM also prevented the increase in the mRNA expression levels of the osteochondrogenic markers *Msx2* and *Sox9*, without modifying *SMMHC* and *Runx2* mRNA expression levels (Fig. 1C). In addition, AFC increased the level of the calcification inhibitor PPi in the culture supernatant of aortic rings cultured under high-phosphate conditions (Fig. 1D). This was observed while the increase in TNAP activity under high-phosphate conditions was not modified by 1 µM AFC (Supplementary Fig. 2).

Efficient inhibition of sEH-P activity, with no change in sEH-H activity, by 1 µM AFC was confirmed by a decrease in the 18:1 MAG-to-18:1 LPA ratio without changes in 14,15-DHET-to-14,15-EET ratio in the culture supernatant (Supplementary Figs. 3A and 3B). The anticalcifying effect of sEH-P inhibition was confirmed using another recently developed, specific sEH-P inhibitor, SWE101 (Fig. 1E) [16], and this effect appears even greater than that obtained with AFC at the same concentration. Moreover, SWE101 was used to illustrate the prevention of the increased Msx2 protein expression under high-phosphate conditions (Supplementary Fig. 4). In addition, aortic rings isolated from sEH-P knock-in (KI) rats, which lack of sEH-P activity [9], were less prone to calcify compared to aortic rings from control wild-type (WT) rats when cultured under similar high-phosphate conditions (Fig. 1F).

### Endothelium-dependent but NO-independent mechanisms are involved in the effects of sEH-P inhibition

**Fig. 2:**
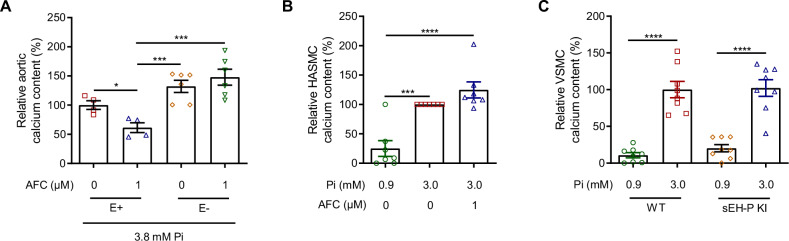
Prevention of calcification by sEH-P inhibition requires the vascular endothelium. Relative calcium content of intact (E+) and deendothelialized (E-) aortic rings cultured during 7 days under high-phosphate (3.8 mM Pi) conditions, in absence and in presence of the sEH-P inhibitor *N*-acetyl-S-farnesyl-L-cysteine (AFC; **A**). Relative calcium content of human aortic smooth muscle cells (HASMC) cultured during 14 days under normal (0.9 mM Pi) and high-phosphate (3.0 mM Pi) conditions, in absence and in presence of 1 µM AFC (**B**) and of aortic vascular smooth muscle cells (VSMC) isolated from WT and sEH-P KI rats cultured during 14 days in 0.9 and 3.8 mM Pi (**C**). Mean and SEM values are shown and group effect was determined by one-way *ANOVA* with Bonferroni’s post hoc test. *P < 0.05, ***P < 0.001, ****P < 0.0001.

Subsequently, the mechanism possibly involved in the prevention of calcification associated with sEH-P inhibition was explored. Deendothelialization of the aortic rings prevented the effect of AFC on calcium deposition (Fig. 2A). In addition, AFC did not affect high-phosphate-induced calcification of primary human aortic smooth muscle cells (HASMC) and aortic SMC isolated from KI and WT rats calcified similarly when cultured under high-phosphate conditions (Fig. 2B, C). These results demonstrate that the endothelial cells play a major role in the anticalcifying effect associated with sEH-P inhibition. Although an interaction between sEH-P and endothelial NO-synthase (eNOS) has been previously suggested in vitro [17, 18], the concentration of the NO metabolite nitrite in the culture supernatants of aortic rings was not affected by AFC, and the addition of the eNOS inhibitor Nω-Nitro-L-arginine (L-NNA) did not reverse the impact of AFC on aortic calcium deposition (Supplementary Fig. 5A, B). These results suggest that eNOS is not involved in the anticalcifying effect of AFC.

### sEH-P metabolizes intracellular PPi

**Fig. 3:**
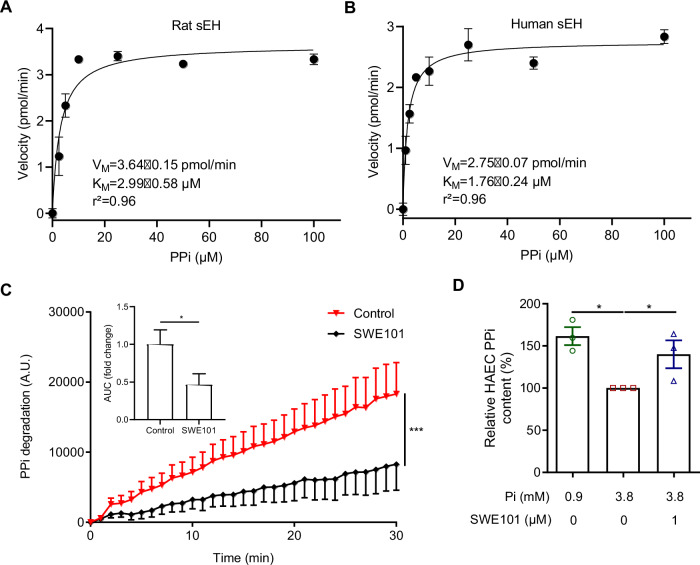
sEH-P contributes to the intracellular degradation of PPi. Kinetic analysis of rat (**A**) and human (**B**) recombinant sEH towards pyrophosphate anions (PPi; *n* = 3). The kinetic constants (*K*_M_ and *V*_M_) were calculated by non-linear fitting of the Michaelis equation using the enzyme kinetic module of using GraphPad Prism software 8.0.2. Time course of 10 µM PPi degradation by human recombinant sEH in absence and in presence of the sEH-P inhibitor SWE101 at 1 µM (**C**). Relative PPi content in HAEC cultured during 3 days under normal (0.9 mM Pi) and high-phosphate (3.8 mM Pi) conditions, in absence and in presence of 1 µM SWE101 (**D**). Mean and SEM values are shown and group effect was determined by one-way *ANOVA* or repeated measured *ANOVA* with Bonferroni’s post hoc test.

Based on the previously demonstrated efficiency of sEH-P to metabolize molecules containing a pyrophosphate moiety to a mono-phosphate [8], and the increase in PPi associated with sEH-P inhibition, without change in TNAP activity, this compound was tested as a sEH-P substrate. Kinetic experiments were performed with recombinant sEH. The rat and human sEH were efficient in hydrolyzing PPi, and this hydrolysis was reduced by sEH-P inhibition with SWE101 (Fig. 3A–C). Surprisingly, the specific constant (*k*_cat_/*K*_M_) for the human sEH (15.7.10^−3^ s^−1^ µM^−1^) indicates that PPi is converted by sEH-P as fast as LPA [8]. According with these results, the decrease in PPi content in immortalized human aortic endothelial cells (HAEC) cultured under high-phosphate conditions was prevented when sEH was inhibited using SWE101 (Fig. 3D).

Altogether, these results show that the anticalcifying effect of sEH-P inhibition is due to a reduction in the degradation of PPi produced by endothelial cells.

### sEH-P inhibition prevents vascular calcification and cardiac remodeling in rats with CKD

**Fig. 4:**
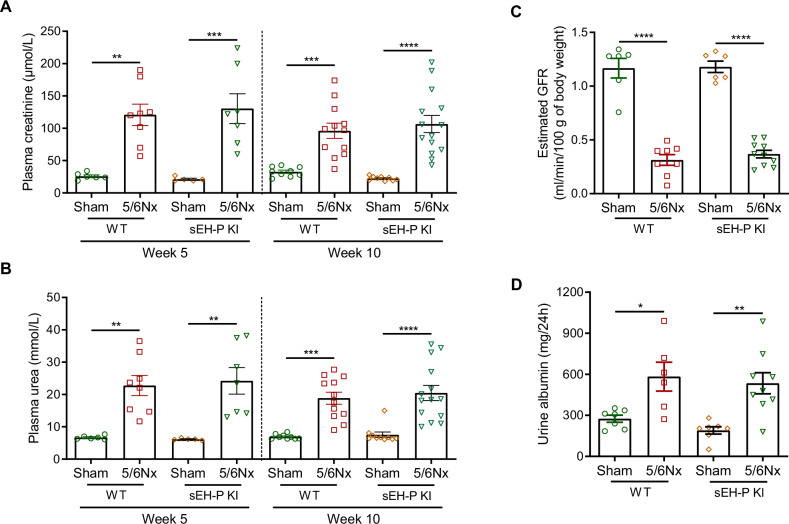
Genetic inactivation of sEH-P did not affect the development of CKD. Plasma creatinemia (**A**), urea nitrogen (**B**), estimated glomerular filtration rate (GFR, **C**) and 24h-urinary albumin (**D**) determined 5 and/or 10 weeks after surgery in WT and sEH-P KI sham-operated and 5/6 nephrectomized rats (5/6Nx). Mean and SEM values are shown and group effect was determined by one-way *ANOVA* with Bonferroni’s post hoc test. *P < 0.05, ****P < 0.0001.

To assess whether sEH-P inhibition is effective in preventing vascular calcification in vivo, CKD was induced by subtotal nephrectomy in sEH-P KI rats and control WT rats. As expected, sEH-P activity was markedly reduced in the renal tissue of knock-in rats, without significant change in sEH-H activity (Supplementary Fig. 6). Over the 10-week period following surgery, the mortality rate was similar in sEH-P knock-in rats and control rats (Supplementary Fig. 6). CKD developed similarly in both strains, as shown by the parallel increases in plasma creatine and urea, the reduction of glomerular filtration rate, estimated using transdermal measurement of FITC-sinistrin clearance, and the increased albuminuria in nephrectomized WT and KI rats as compared to sham-operated rats (Fig. 4A–D). In addition, the degree of polyuria and polydipsia was similar in both strains (Supplementary Fig. 8A and 8B), as were histological lesions (Supplementary Fig. 9).

**Fig. 5:**
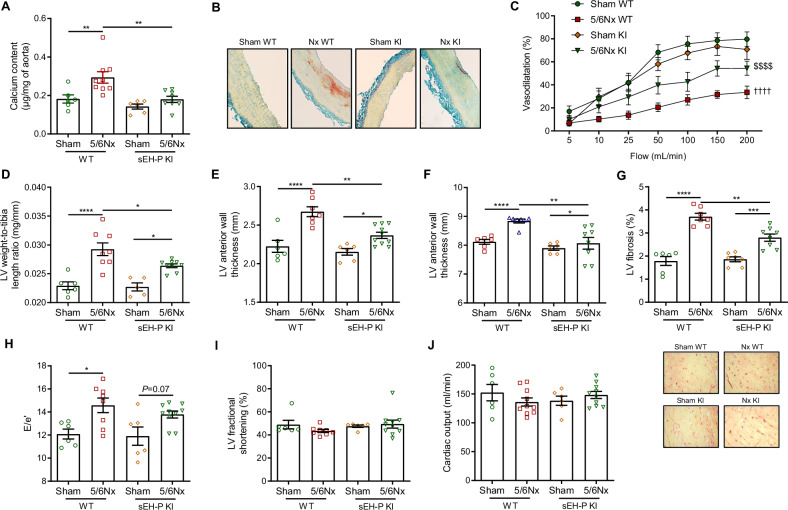
Genetic inactivation of sEH-P prevents vascular calcification and cardiac remodeling in CKD rats. Calcium content (**A**) and representative Alizarin red staining (**B**) of aortas, flow-mediated dilatation of second mesenteric resistance artery (**C**), left ventricular (LV) weight-to-tibia length ratio (**D**), LV anterior wall thickness (**E**), LV end-diastolic diameter (**F**), LV ventricular fibrosis and representative Sirius red staining (**G**), LV E/e’ ratio (**H**), LV fractional shortening (**I**) and cardiac output (**J**) determined in WT and sEH-P KI sham-operated and 5/6 nephrectomized rats (5/6Nx). Mean and SEM values are shown, and group effect was determined by one-way *ANOVA* (**A**, **D**–**J**) or repeated measures ANOVA (**C**) with Bonferroni’s post hoc test. *P < 0.05, **P < 0.01, ****P < 0.0001. ^††††^*P* < 0.0001 vs. WT sham. *P* < 0.0001 vs. WT 5/6Nx.

In this context and despite the absence of change in the blood calcium-phosphate product (Supplementary Fig. 9), nephrectomized WT rats displayed aortic calcification. This was evidenced by increased aortic calcium content and Alizarin red staining compared to sham-operated rats (Fig. 5A, B). Notably, the development of aortic calcification was fully prevented in the sEH-P knock-in rats (Fig. 5A, B). In addition, the degree of vascular dysfunction associated with CKD was lesser in sEH-P knock-in rats compared to WT rats (Fig. 5C). At the cardiac level, left ventricular (LV) hypertrophy and dilatation were reduced in nephrectomized sEH-P knock-in rats compared to nephrectomized WT rats. This was demonstrated by decreases in the LV weight-to-tibia length ratio and in end-diastolic LV wall thickness and LV diameter (Fig. 5D–F). In addition, sEH-P KI partially prevented the development of LV fibrosis induced by nephrectomy (Fig. 5G). In contrast, sEH-P knock-in did not appear to prevent the development of cardiac diastolic dysfunction, as illustrated by the increase in the E/e’ ratio with nephrectomy (Fig. 5H). There was no change in systolic function, i.e., cardiac output and fractional shortening (Fig. 5I, J).

## Discussion

The major finding of the present study is that sEH-P promotes vascular calcification through the degradation of PPi and its inhibition offers protection against cardiovascular abnormalities in experimental CKD.

First, ex vivo experiments demonstrated that pharmacological or genetic inhibition of sEH-P reduces the calcification of rat aortic rings cultured under high-phosphate conditions. This was associated with prevention of the increase in mRNA expression levels of *Msx2* and *Sox9* induced by high phosphate, indicating a reduction in the osteochondrogenic transition of VSMCs. The osteogenic factor MSX2 and the chondrogenic factor SOX9 are known to exert opposite effects on *Runx2* expression, which may help explain the absence of a significant effect of sEH-P inhibition on *Runx2* levels [19]. In fact, inhibition of sEH-H has previously been reported to enhance *Runx2* expression [10], while inhibition of sEH-P has been proposed to downregulate *Msx2* and *Sox9*, which may act upstream of *Runx2*, at least in the context of bone development [19]. Although this has to be confirmed at the protein level, one can hypothesize that the sEH phosphatase domain may exert its deleterious role at an early stage of the vascular calcification process by modulating upstream transcription factors, whereas the hydrolase domain may play a more prominent role at later stages by directly influencing *Runx2*. Supporting this, no change in *Msx2* and *Sox9* expression levels was observed in endarterectomy specimens of calcified carotid plaques compared to the adjacent non-calcified segments, while *Runx2* expression was markedly increased and inversely correlated with sEH gene expression [10], suggesting a greater impact of the hydrolase domain at this advanced stage of the disease.

Second, the absence of a preventive effect of sEH-P inhibition on deendothelialized aortic rings and on human and rat VSMC strongly supports the hypothesis that endothelium-dependent mechanisms are involved. This aligns with previous findings showing that sEH-H metabolizes the procalcifying endothelial factors EETs [10]. Our results excluded the involvement of the eNOS pathway, which has previously been shown to prevent vascular calcification [20, 21], contrasting with observations in endothelial cells stimulated with VEGF or statins [17, 18]. We then investigated the role of PPi, an endogenous inhibitor of the formation and growth of phosphate-calcium crystals, primarily hydroxyapatite [22–24]. PPi was identified as a natural substrate of sEH-P. The prevention of high-phosphate-induced PPi degradation and release by endothelial cells through sEH-P inhibition may represent a key mechanism underlying its anticalcifying effects. This should translate into an increase in extracellular PPi, as suggested by the elevated PPi levels observed in the culture supernatant of aortic rings treated with an sEH-P inhibitor, without a significant change in TNAP activity, thereby reducing calcium crystal formation. In addition, these findings suggest that sEH is the main enzyme involved in the catabolism of intracellular PPi, while TNAP mediates its degradation at the extracellular level, and that both pathways play a key role in regulating the Pi/PPi balance and vascular mineralization [24]. Interestingly, two substrates of sEH-P, PPi and LPA, are formed by enzymes of the same family: the ectonucleotide pyrophosphatases/phosphodiesterases (eNPP1 and eNNP2, the latter also termed autotaxin). Although a similar role for eNPP1 and eNPP2 at the intracellular level remains unclear, many other enzymes and enzymatic pathways contribute to the production of PPi and LPA in cells [25, 26]. Finally, we cannot exclude the possibility that, in addition to inhibiting calcification formation, PPi may also directly modulate osteochondrogenic gene expression, as recently reported in bone cells [27]. In addition, the modulation of PPi levels may not be the sole endothelium-dependent mechanism involved in the preventive effects of sEH-P inhibition against calcification, given its previously demonstrated impact on the signaling of intracellular lipid mediators, particularly the LPA/PPARγ pathway [9], which may modulate cell function, including inflammation.

Furthermore, in the absence of modification to the degree of renal alterations induced by subtotal nephrectomy, the comparison of WT and sEH-P KI rats demonstrated that the loss of function of sEH-P efficiently prevents the development of vascular calcification in vivo. Thus, part of the decrease in aortic calcification observed in sEH knockout mice fed with a high adenine and phosphate diet may be at least partly related to the suppression of sEH-P activity, rather than solely due to the previously-demonstrated interaction with Sirt3 [11]. In addition, we cannot exclude the possibility that modifications in local tissue phosphate levels or in phosphate transporters may have contributed to the prevention of aortic calcification in CKD rats.

Furthermore, our study revealed that sEH-P inhibition prevented the development of cardiac remodeling induced by CKD, with reductions in LV dilatation and fibrosis. The lack of improvement in diastolic function should be confirmed but may be related to persistent microvascular dysfunction limiting LV relaxation, intrinsic cardiomyocyte stiffening, and/or residual cardiac volume overload, all of which could mask improvements in ventricular compliance. We previously demonstrated that sEH-P KI rats are protected against the development of cardiac ischemia–reperfusion injury, and consistent with these results, KI rats were also protected against cardiac remodeling in the context of obesity and insulin resistance; however, once again, the improvement in diastolic function was not significant [9]. Given the established role of sEH-H in the regulation of cardiac function and remodeling, additional experiments are warranted to better define the specific contribution of sEH-P at this level.

Altogether our results show that sEH-P contributes to the development of vascular calcification by degrading the mineralization inhibitor PPi in endothelial cells. Thus, it appears that sEH plays a dual role in the vascular calcification process, with its phosphatase and hydrolase domains performing opposite roles. The hydrolase domain has been previously shown to be protective, particularly by limiting EETs-mediated increase in TNAP activity and PPi degradation [10]. Importantly, the preventive effect of sEH-P against vascular calcification we evidence in the present work in a murine model of CKD was also associated with an improvement in vascular function and cardiac remodeling, further highlighting the interest of this therapeutic approach in this pathophysiological context. Beyond CKD, ectopic calcification is a common and clinically relevant feature of several other diseases, including atherosclerosis, diabetes-associated vascular disease, calcific aortic valve stenosis, and rare inherited disorders such as pseudoxanthoma elasticum and generalized arterial calcification of infancy. In all these conditions, abnormal calcification contributes to progressive tissue stiffening, impaired organ function, and increased cardiovascular risk, yet therapeutic options remain limited. Further development of pharmacological inhibitors of sEH-P is therefore warranted, both to confirm their capacity to prevent ectopic calcification in broader pathophysiological contexts and to establish their potential as a strategy to manage cardiovascular complications in kidney diseases and other calcification-prone conditions.

## Materials and methods

An expanded detailed description of this section is available in the Supplementary Materials.

### Animal ex vivo and in vivo experiments

Experiments were performed in 12-weeks-old male wild type (WT; *n* = 85, 31 for in vivo and 54 for ex vivo experiments) Sprague-Dawley rats and rats with a specific inactivation of sEH-P activity (*n* = 52, 36 for in vivo and 16 for ex vivo experiments), generated using the CRISPR-Cas9 technology as previously described [9]. The sample size was determined based on previous ex vivo and in vivo experiments [9, 10, 28], and we combined analyses of tissues and culture supernatants ex vivo with functional and biological investigations in vivo to minimize the use of animals. All animal studies were approved by the Animal Ethics Committee (CENOMEXA #24107) and performed in accordance with the care and use of laboratory Animal’s guide.

For ex vivo mineralization assays, rats were euthanized by intraperitoneal injection of pentobarbital (120 mg/kg), and the thoracic and abdominal aortas were dissected as previously described [10]. After removal of fat and connective tissues, the vessels were cut into 2- to 3-mm rings and randomly placed in regular medium (DMEM 6546, Sigma-Aldrich, Saint-Louis, MO), containing 0.9 mM inorganic phosphate (Pi), or in high-phosphate DMEM with 3.8 mM Pi, supplemented with 10% FBS at 37 °C in 5% CO_2_ for 7 consecutive days, which is required to induce calcification of aortic rings [10]. The medium was changed on the 3rd and 6th days. The deendothelialization of aortic rings was performed by gently rubbing the intimal surface of the aortic rings with a wooden stick, as previously validated [10].

For in vivo experiments, WT and sEH-P KI rats were randomly submitted to either a two-step surgical procedure to induce CKD or a sham surgery [27]. The first step of the surgical procedure consisted in the ligation of the upper branch of the left kidney artery, followed by a cauterization of the lower pole of the left kidney, leading to 2/3 of non-functioning left kidney. One week later, the right kidney was removed, inducing 5/6 nephrectomy (5/6 Nx). Sham animals underwent laparotomy and manipulation of both kidneys, without removing them, before wound closure. An assessment of cardiovascular and renal parameters was performed 10 weeks after surgery [28].

### Cell experiments

Primary HASMC were isolated from aortic lesion-free explants as previously described [10]. Human arterial tissues were obtained from patients undergoing aortic surgery, who gave written informed consent, and studies were conducted in accordance with the Principles of Good Clinical Practice and the Declaration of Helsinki and approved by the local ethical committees (Protocol number 2009-19). For mineralization assays, primary HASMC were cultured between P3 and P8 (100,000 cells/well; 6-well plates) in regular (0.9 mM Pi) or high-phosphate DMEM (3 mM Pi) supplemented with 1% FBS at 37 °C in 5% CO_2_ for 14 consecutive days. The medium was changed every 2 to 3 days.

Immortalized HAEC (ATCC CRL-4052) were cultured in 12-well plates with endothelial cell growth kit-VEGF (ATCC PCS-100-041) and supplemented by 10 units/mL penicillin and 10 μg/mL streptomycin (P4333, Sigma-Aldrich) at 37 °C in humidified atmosphere with 5% CO_2_. Immortalized HAEC were cultured in regular (0.9 mM) or high-phosphate (3.8 mM Pi) for up to 3 days.

### Statistical analysis

Statistical analyses were conducted using GraphPad Prism software 8.0.2. Group effects were determined by *ANOVA* or repeated measures *ANOVA* with Bonferroni’s post hoc test. Parameters obtained in all living animals by non-blinded investigators were included into analysis. Data are presented as mean ± SEM with individual values. Two-sided *P* values less than 0.05 were considered statistically significant.

## Supplementary information

Supplements

## Data Availability

The current work does not contain big data sets or self-designed algorithms. The authors will share any detailed information about any of the animal models or analytical procedures with anyone who wishes to use these models or procedures in their research. Additionally, individual data can be provided for further scientific analysis upon request by contacting the corresponding author, JB, at jeremy.bellien@chu-rouen.fr.
