# Supplementary material for: The phosphatase activity of soluble epoxide hydrolase regulates vascular calcification through the metabolism of pyrophosphate anions
Source: Cell Death Dis. 2025 Dec 27;17(1):143. doi: 10.1038/s41419-025-08390-6 (PMC12847717; doi:10.1038/s41419-025-08390-6)
Supplement: Supplementary file 1 — Supplements [file 41419_2025_8390_MOESM1_ESM.docx]

**The Phosphatase Activity of Soluble Epoxide Hydrolase Regulates Vascular Calcification Through the Metabolism of Pyrophosphate Anions: Implications for Chronic Kidney Disease**

Messaoudi et al

**SUPPLEMENTARY MATERIALS**

**SUPPLEMENTARY METHODS**

***In vitro* and *ex vivo* experiments**

Pharmacological agents

Inhibition of the phosphatase domain of soluble epoxide hydrolase (sEH-P) was first obtained using *N*-Acetyl-S-farnesyl-L-cysteine (AFC; 63270, Cayman Chemical Company). AFC has an IC50 value against sEH phosphatase of around 0.84 ± 0.9 µM and nonspecific effects *i.e.*, inhibition of methyl transferase enzymes, at a concentration higher than 20 µM.^1,2^ To confirm the results obtained with AFC, complementary experiments were performed with the recently-validated more specific sEH-P inhibitor 4-(4-(3,4-Dichlorophenyl)-5-phenyloxazol-2-yl)butanoic acid (SWE101: 1 µM) that exhibits an IC50 value of 0.058 ± 0.005 µM.^3^ The role of NO-synthase was assessed using co-treatment of aortic rings with Nω-Nitro-L-arginine (L-NNA: 100 µM; Sigma-Aldrich).

LC-MS/MS analysis

To monitor the impact of AFC on sEH enzymatic activities, 14,15-EET, 14,15-DHET, oleoyl-L-α-lysophosphatidic acid (oleoyl-LPA) and its metabolite 1-oleoyl-*rac*-glycerol (OG) were quantified in the aortic culture supernatant at day 6 by liquid chromatography coupled to tandem mass spectrometry as previously descrived.^4^ 14,15-EET and 14,15-DHET analysis was performed after protein precipitation, lipids extraction and saponification. Briefly, 300 µL of culture supernatant were spiked with 10 µL of internal standards (14,15-EETd_8_ and 11,12-DHETd_11_ at 200 ng/mL) and 1mL of methanol was added for protein precipitation. After centrifugation, the supernatant was collected and lipids were extracted with dichloromethane after acidification with formic acid. The organic layer was evaporated to dryness under a gentle stream of nitrogen. The crude extract was saponified with sodium hydroxide at +90 °C for 20 min to release fatty acids into their free form. Then, the sample was acidified with formic acid and EETs and DHETs were extracted with a second extraction. The sample was once again evaporated to dryness and reconstituted in 100 µL of methanol. Chromatographic separation was performed on a Kinetex C18 column (2.6-μm particle size, 50-mm length × 3-mm inner diameter). The autosampler temperature was set at 8 °C, the column oven at 30 °C, the injected volume was 20 μL, and the flow rate was 600 μL/min. The mobile phase was 0.2% formic acid in MeOH (solvent A) and 2 mM ammonium formate with 0.2% formic acid in water (solvent B). The elution was started with 95% B (0-0.5 min), 95-20% B (0.5-1 min), 20-10% B (1-4 min), 10-5% B (4-5 min), 5-95% B (5-6 min), 95% B (6-7.5 min). The following multiple reaction monitoring (MRM) transitions m/z 319.1 to m/z 219.1 and m/z 337.1 to m/z 207.1 in negative ion mode were used to detect 14,15-EET and 14,15-DHET respectively.

For the determination of oleoyl-LPA and OG, 200µL of methanol (MeOH) was added to 100 µL of culture supernatant in a 1.7 mL Eppendorf tube. The sample was thoroughly vortexed for 10 seconds and centrifuged at 16,100 x g for 5 min. Chromatographic separation was achieved on a Kinetex C18 column (2.6-μm particle size, 50-mm length × 3-mm inner diameter). The autosampler temperature was set at 8 °C, the column oven at 30 °C, the injected volume was 20 μL, and the flow rate was 500 μL/min. The mobile phase was 1% acetic acid in MeOH (solvent A) and 10 mM ammonium acetate with 1% acetic acid in water (solvent B). The elution was started with 30% B (0-0.5 min), 30-1% B (0.5-3 min), 1% B (3-9 min), 1-30% B (9-10 min), 30% B (10-12 min). The following MRM transitions m/z 435 to m/z 152.9 in negative ion mode and m/z 357.2 to m/z 265.1 in positive ion mode were used to detect oleoyl-LPA and OG respectively.

Chemiluminescence assay

The concentrations of the NO metabolite nitrite were determined in the culture supernatant of aortic rings at day 6 using a tri-iodide/ozone-based chemiluminescence assay.^5^ Thawed culture supernatant (250 µL) was injected into a glass purge vessel containing acidified tri-iodide solution, at room temperature, and actively purged with nitrogen in-line with a gas-phase chemiluminescence NO analyzer (NO Analyzer 280, Sievers). The tri-iodide solution rapidly reduces NO metabolites, mainly nitrite (>95% in plasma), to NO gas, which is measured by its gas-phase chemiluminescent reaction with ozone:

NO + O_3_ 🠆 NO_2_^*^ + O_2_

NO_2_^*^ 🠆 NO_2_ + hυ

The excited state of nitrogen dioxide emits light, which is then detected by a photomultiplier tube. Quantification of nitrite in a sample involves the conversion of the NO Analyzer signal into actual concentration using a calibration curve drawn from standard nitrite solutions prepared in phosphate buffer.

Viability assay

Methylthiazolyldiphenyl-tetrazolium bromide (MTT; M5655, Sigma-Aldrich), a yellow tetrazole reduced to purple formazan in living cells, was used to determine the viability of aortic rings. The MTT substrate was prepared in a PBS solution at a final concentration of 0.05 mg/mL. Aortic rings were incubated in MTT solution for one at 37 °C and 5% CO_2_. The MTT solution was then removed and the aortic rings were left 2 hours at room temperature. DMSO was added to dissolve formazan and changes in absorbance at 570 nm, which is directly proportional to the quantity of viable cells, were measured.

Quantification of calcium deposition

Aortic segments or human VSMC were washed once with PBS and then decalcified with 0.6 N HCl overnight at 4 °C. The calcium content in the HCl supernatant was colorimetrically analyzed with the o-cresolphthalein complexone method.^6^ Briefly, a solution of ortho-cresolphtalein complexone at 300 µM (P5631, Sigma-Aldrich) is prepared with 8-hydroxyquinoline 16 mM (252565, Sigma-Aldrich) in 2-amino-2-methyl-1-propanol 0.5 M distilled water solution (pH=10.5; Sigma-Aldrich) and heated for two hours. For the quantification, 25 µL of HCl supernatants are added to 150 µL of ortho-cresolphtalein solution in 96-well plates. Plates are shacked for 15 min at 500 RPM and the absorbance is then measured at 565 nm. Calcium content in aortic rings was corrected by aortic dry weight with aortas dried overnight at 37 °C.

# Pyrophosphate (PPi) assays

PPi concentrations in the culture supernatant of aortic rings were determined using a fluorogenic pyrophosphate sensor following the manufacturer instructions (MAK168 Sigma Aldrich).^6^ Briefly, samples were diluted (1/5 assay buffer) and 20 µL were mixed with 20 µL of PPi sensor. Fluorescence was read within 15 min using microplate reader (Flexstation, Molecular Devices; λex = 316/λem = 456 nm). After background signal (no PPi) removal, the concentration was determined from a PPi standard curve (10^-7^ to 10^-4^ M).

The hydrolysis of PPi by sEH was measured using the PiPer Pyrophosphate assay kit from Invitrogen (Waltham, PA; catalog number MP22062). The recombinant human and rat sEH were obtained and purified as described.^7^ Because the presence of phosphate buffer affects dramatically the assay kit, the buffer in which the enzymes are purified into was changed from a sodium phosphate buffer (pH 7.4; 0.1 M), to a Bis-Tris/HCl buffer (pH 7.0; 25 mM). In 96-well microplates, 50 µL of diluted enzymes or buffer for control were mixed with 50 µL of Amplex® Red reagent/inorganic pyrophosphatase/maltose phosphorylase/maltose/glucose oxidase/HRP/substrate working solution (see assay kit for details; [E]final: Human sEH 17 nM, Rat sEH 102 nM). The mixtures were incubated at 37 °C for 5 minutes. The enzymatic reactions were started by the addition of 1 µL of a 100-fold concentrated PPi solution in buffer ([S]final: 0 to 100 µM). The reaction was followed at 37 °C by measuring the appearance of resorufin by fluorescence (excitation: 540 nm; emission: 590 nm) kinetically for 30 minutes (measurement every 30 seconds) on a Spectramax M2 plate reader (Molecular Devices, San Jose, CA). Reactions were run in hexa-plicates (n =6). Because the reaction took few minutes to reach steady state, the first 10-minutes of reaction were not used to calculate the velocity of the enzyme. The non-linear regression of the velocity in function of the [S] were used to calculate the kinetic constants using SigmaPlot version 14.0 (Systat Software, Chicago, IL). Results are average +/- SD of three separate measurements.

Immortalized human aortic endothelial cells (HAEC; ATCC CRL-4052) were challenged with 3.8 mM Pi for up to 3 days and cultured in the presence or not of the sEH-P inhibitor SWE101 at 1µM. The PPi intracellular levels were tested in a 96-well plate using a commercial pyrophosphate assay kit (EnzChek™, Invitrogen). PPi levels were normalized to the intracellular protein concentration.

Tissue-nonspecific alkaline phosphatase (TNAP) activity

TNAP activity in the culture supernatant of aortic rings was measured, as the hydrolysis of pnitrophenyl phosphate (pNPP), using a commercially available kit (AP0100, Sigma-Aldrich). Briefly, a pNPP solution 0.67 M was prepared in ultrapure water. In a 96 well-plate, 5 µl of cell culture supernatants were added to 240 µl of reaction buffer kit and 5 µl of pNPP solution. A blank well and a control enzyme well were used. Plates were incubated at 37°C with the absorbance at 405 nm measured each minute during 10 minutes and TNAP activity was calculated using a molar extinction coefficient of 18.75 mM/cm.

# Histological assessment of the osteochondrogenic differentiation

Samples were cryopreserved in OCT (Optimum Cutting Temperature, O.C.T Compound, Sakura^®^ Finetek). Seven μm-thick cross sections were obtained perpendicularly to the vessels (Leica^®^ CM 1850 Cryostat). Briefly, the slides were stained 2 minutes with Alizarin Red S solution (40 mmol/l, pH = 4.2, at room temperature) to observe specifically calcium ions. Slides were then immersed successively in 3 baths (100% acetone, acetone-toluene (1:1) and 100% toluene) to dehydrate them completely. Von Kossa staining (VK) was used to identify divalent ions. The slides were fixed by ethanol with 2 successive baths for 5 minutes. A 1% silver nitrate solution was added in the dark for 30 minutes and slides were then rinsed with water. Sodium thiosulfate (5%) was added for 5 minutes, rinsed in water and Kernechtrot counterstain was added (0.2% Kernechtrot in aqueous solution, 5% aluminium sulfate) for 5 minutes. The slides were washed with water and dehydrated with 100% EtOH and toluene before observations.

Quantitative RT-PCR

Total RNA was extracted using the RNA isolation kit RNeasy^®^ Mini Kit (Qiagen). Then 500 ng RNA was reversed transcribed into cDNA using the ‘High-Capacity cDNA Reverse Transcription Kit' (Applied Biosystems) according to the manufacturer's instructions. Primers for the osteochondrogenic transcription factor genes Runt-related transcription factor 2 (Runx2), Msh homeobox 2 (Msx2), and sex determining region Y-box 9 (Sox9), the contractile marker smooth muscle myosin heavy chain (SMMHC), and GAPDH were from Eurogentec (See Supplementary Table 1). The quantitative PCR was performed on Biorad CFX connect RealTime System using SYBR Green Master Mix and RNA expression levels of the different genes were corrected for GAPDH.

Western blot

Western blotting was performed on aortic rings cultured as previously described for *ex vivo* mineralization assays in absence and in presence of the sEH-P inhibitor SWE101 at 1µM. In brief, aortic rings were washed twice with PBS, and proteins were extracted using RIPA buffer (Sigma-Aldrich) supplemented with protease inhibitor cocktail (Roche Diagnostics) and phosphatase inhibitor cocktail (Calbiochem). Protein concentration was determined using the Pierce™ BCA Protein Assay Kit (Pierce). A quantity of 40 µg of protein from each sample was separated by sodium dodecyl sulfate–polyacrylamide gel electrophoresis (SDS-PAGE) and transferred to a nitrocellulose membrane. Membrane was washed with Tris-buffered saline containing 0.05% Tween-20 (TBS-T) and blocked with 5% BSA + 5% dried milk in TBS-T. Primary antibodies for Msx2 (1 : 1000 ; Invitrogen) and GAPDH (1 : 10000 ; Invitrogen) were used to incubate the membrane ON at 4°C. Detection was performed using appropriate horseradish peroxidase–conjugated secondary antibodies (Jackson ImmunoResearch, West Grove, PA, USA; 1:10,000), and signals were developed with SuperSignal™ Western Pico Chemiluminescent Substrate (Bio-Rad).

***In vivo* experiments**

Renal Parameters

Water intake and 24-h urine volume were obtained 5 and 10 weeks after surgery in rats placed in metabolic cages. Plasma creatinine, urea and the calcium-to-phosphorus product were assessed 5 and 10 weeks after surgery thanks to 100 µL-retro-orbital blood collection and aortic blood sampling respectively in rats anesthetized with 1-2% isoflurane. Transcutaneous measurement of glomerular filtration rate (GFR) was performed 9 weeks after surgery by using fluorescein-isothiocyanate-labelled sinistrin in freely moving rats as previously described.^8^ The remnant kidney of nephrectomized rats and the left kidney of sham-operated rats were removed and weighted at sacrifice. The kidney weight was normalized to tibia length. In addition, Masson's trichrome staining of paraffin‐embedded kidney sections was used for semi‐quantitative scoring of glomerular, tubular injury, interstitial inflammation and interstitial fibrosis as previously described.^9^

Cardiac parameters

Echocardiography was carried out 9 weeks after surgery under light anesthesia (1–2% isoflurane) using a Vivid 7 ultrasound echograph (GE Healthcare, Buc) equipped with a M12L linear probe operating at 14 MHz and fitted out with Echopac PC software (GE Medical Systems). All echocardiography studies were performed and analyzed by a single operator. Briefly, a two-dimensional parasternal long axis view of the left ventricle was obtained at the level of the papillary muscle, in order to record M-mode tracings. Left-ventricular (LV) end-diastolic (EDD) and systolic diameters (ESD), and end-diastolic anterior LV wall thickness were measured by the American Society of Echocardiology leading-edge method from at least 3 consecutive cardiac cycles. LV fractional shortening (FS) was calculated from the variation in LV diameters as FS (%)=((LVEDD-LVESD)/LVEDD)×100. In addition, LV outflow velocity was measured by pulsed‐wave Doppler, and cardiac output was calculated as CO = aortic VTI x [π x (LV outflow diameter/2)2] x heart rate, where VTI is velocity–time integral, as described previously.^9^ Doppler measurements were made at the tip of the mitral leaflets for diastolic filling profiles in the apical four-chamber view, allowing to determine the E/e’ ratio (mitral inflow E wave/e’ tissue Doppler mitral annulus velocity), as estimate of diastolic function. Rat hearts were removed and dissected. The LV weight was normalized to tibia length. In addition, Sirius red staining of LV cryosections was used for collagen determination.

Vascular parameters

At sacrifice, endothelium-dependent flow-mediated dilatation was assessed on second mesenteric resistance artery segment. Briefly, the mesentery was removed and placed in cold oxygenated Krebs buffer. A 2–3 mm segment of third mesenteric resistance artery segment was isolated and mounted on an arteriograph (DMT, Denmark). Vessels were pre-constricted using 10^-5^ M phenylephrine before assessing the dilatory response to stepwise increase in intraluminal flow (0, 5, 10, 25, 50, 100, 150 and 200 μL/min). In addition, the thoracic aorta was removed and cut in half. One segment was washed once with PBS and then decalcified with 0.6 N HCl overnight at 4°C. The calcium content in the HCl supernatant was colorimetrically analyzed with the o-cresolphthalein complexone method cresol-phtalein method.^6^ Calcium content in aortic rings were corrected by aortic dry weight with aortas dried overnight at 37°C. In addition, calcium deposition was evaluated on the second thoracic segment using 7 μm-thick histological slices stained with Alizarin red.

# References

1. Enayetallah AE, Grant DF. Effects of human soluble epoxide hydrolase polymorphisms on isoprenoid phosphate hydrolysis. Biochem Biophys Res Commun. 2006;341:254-60.
2. Volker C, Miller RA, McCleary WR, Rao A, Poenie M, Backer JM, Stock JB. Effects of farnesylcysteine analogs on protein carboxyl methylation and signal transduction. J Biol Chem 1991;266:21515-22.
3. Kramer JS, Woltersdorf S, Duflot T, Hiesinger K, Lillich FF, Knöll F, Wittmann SK, Klingler FM, Brunst S, Chaikuad A, Morisseau C, Hammock BD, Buccellati C, Sala A, Rovati GE, Leuillier M, Fraineau S, Rondeaux J, Hernandez-Olmos V, Heering J, Merk D, Pogoryelov D, Steinhilber D, Knapp S, Bellien J, Proschak E. Discovery of the First in Vivo Active Inhibitors of the Soluble Epoxide Hydrolase Phosphatase Domain. J Med Chem. 2019;62:8443-8460.
4. Leuillier M, Duflot T, Ménoret S, Messaoudi H, Djerada Z, Groussard D, Denis RGP, Chevalier L, Karoui A, Panthu B, Thiébaut PA, Schmitz-Afonso I, Nobis S, Campart C, Henry T, Sautreuil C, Luquet SH, Beseme O, Féliu C, Peyret H, Nicol L, Henry JP, Renet S, Mulder P, Wan D, Tesson L, Heslan JM, Duché A, Jacques S, Ziegler F, Brunel V, Rautureau GJP, Monteil C, do Rego JL, do Rego JC, Afonso C, Hammock B, Madec AM, Pinet F, Richard V, Anegon I, Guignabert C, Morisseau C, Bellien J.CRISPR/Cas9-mediated inactivation of the phosphatase activity of soluble epoxide hydrolase prevents obesity and cardiac ischemic injury. J Adv Res. 2023;43:163-174.
5. Bellien J, Iacob M, Remy-Jouet I, Lucas D, Monteil C, Gutierrez L, Vendeville C, Dreano Y, Mercier A, Thuillez C, Joannides R. Epoxyeicosatrienoic acids contribute with altered NO and endothelin-1 pathways to conduit artery endothelial dysfunction in essential hypertension. Circulation. 2012;125:1266-1275.
6. Varennes O, Mentaverri R, Duflot T, Kauffenstein G, Objois T, Lenglet G, Avondo C, Morisseau C, Brazier M, Kamel S, Six I, Bellien J. The Metabolism of Epoxyeicosatrienoic Acids by Soluble Epoxide Hydrolase Is Protective against the Development of Vascular Calcification. Int J Mol Sci. 2020;21:4313.
7. Tran KL, Aronov PA, Tanaka H, Newman JW, Hammock BD, Morisseau C. Lipid sulfates and sulfonates are allosteric competitive inhibitors of the N-terminal phosphatase activity of the mammalian soluble epoxide hydrolase. Biochemistry. 2005;44:12179-87.
8. Schock-Kusch D, Sadick M, Henninger N, Kraenzlin B, Claus G, Kloetzer HM, Weiss C, Pill J, Gretz N. Transcutaneous measurement of glomerular filtration rate using FITC-sinistrin in rats. Nephrol Dial Transplant. 2009;24:2997-3001.
9. Hamzaoui M, Roche C, Coquerel D, Duflot T, Brunel V, Mulder P, Richard V, Bellien J, Guerrot D. Soluble Epoxide Hydrolase Inhibition Prevents Experimental Type 4 Cardiorenal Syndrome. Front Mol Biosci. 2021;7:604042.
10. Mulder P, Barbier S, Chagraoui A, Richard V, Henry JP, Lallemand F, Renet S, Lerebours G, Mahlberg‐Gaudin F, Thuillez C. Long‐term heart rate reduction induced by the selective I _f_ current inhibitor ivabradine improves left ventricular function and intrinsic myocardial structure in congestive heart failure. Circulation 2004;109: 1674–1679.

**SUPPLEMENTARY TABLE**

**Supplementary Table 1. Primers used for quantitative RT-PCR**

|  | **Forward (5'-3')** | **Reverse (3'-5')** |
| --- | --- | --- |
| GAPDH | TATGATGACATCAAGAAGGTGG | CACCACCCTGTTGCTGTA |
| Runx2 | CCTCTGACTTCTGCCTCTGG | GATGAAATGCCTGGGAACTG |
| Msx2 | CAGAACCGAAGGGCTAAGGCA | GTAGGAGGCGCTGTATATGG |
| Sox9 | GTGCTGAAGGGCTACGACTGG | GCAGATGCGGGTACTGGT |
| SMMHC | CAGTATTTGGCTGTGGTGGC | CATTGCCGAAAGCCTCCAGG |

# SUPPLEMENTARY FIGURES

**Figure S1.** Viability of rat aortic rings cultured during 7 days under high-phosphate conditions (Pi 3.8 mM) in absence and in presence of *N*-acetyl-S-farnesyl-L-cysteine (AFC) at 1 and 10 µM, assessed using a methylthiazolyldiphenyl-tetrazolium assay. Mean and SEM values are shown and group effect was determined by one-way *ANOVA* with Bonferroni's *post* *hoc* test. *****P*<0.0001.

**Figure S2.** Tissue-nonspecific alkaline phosphatase (TNAP) activity assessed by measuring the hydrolysis of p-nitrophenyl phosphate (pNPP) in the supernatants of aortic rings cultured during 7 days under normal (0.9 mM inorganic phosphate, Pi) and high-phosphate (3.8 mM Pi) conditions, in absence and in presence of 1 µM *N*-acetyl-S-farnesyl-L-cysteine (AFC). Mean and SEM values are shown and group effect was determined by one-way *ANOVA* with Bonferroni's *post* *hoc* test. **P*<0.05.

**Figure S3.** Ratio of 18:1 monoacylglycerol-to-18:1 lysophosphatidic acid (18:1 MAG/18:1 LPA; A) and of 14,15-epoxyeicosatrienoic acid-to-14,15-dihydroxyeicosatrienoic acid (14,15-EET-to-14,15-DHET; B) in the supernatants of aortic rings cultured during 7 days under normal (0.9 mM inorganic phosphate, Pi) and high-phosphate (3.8 mM Pi) conditions, in absence and in presence of 1 µM *N*-acetyl-S-farnesyl-L-cysteine (AFC). Mean and SEM values are shown and group effect was determined by one-way *ANOVA* with Bonferroni's *post* *hoc* test. **P*<0.05; ***P*<0.01.

**Figure S4.** Representative Western blot of MSX2 protein expression in aortic rings cultured during 7 days under normal (0.9 mM inorganic phosphate, Pi) and high-phosphate (3.8 mM Pi) conditions, in absence and in presence of 1 µM SWE101. For each condition, thoracic aorta lysates were generated by pooling tissues from 7 animals to obtain sufficient protein. Equal protein loading was confirmed using GAPDH as a loading control. Data are presented qualitatively.

**Figure S5.** Nitrite levels in the supernatants of aortic rings cultured during 7 days under normal (0.9 mM inorganic phosphate, Pi) and high-phosphate (3.8 mM Pi) conditions, in absence and in presence of 1 µM *N*-acetyl-S-farnesyl-L-cysteine (AFC). Relative calcium content of aortic rings cultured in 3.8 mM Pi during 7 days, in absence and in presence of 1 µM AFC alone and associated with the NO-synthase inhibitor L-NA at 10 µM. Mean and SEM values are shown and group effect was determined by one-way *ANOVA* with Bonferroni's *post* *hoc* test. **P*<0.05; ****P*<0.001.

**Figure S6.** Soluble epoxide hydrolase (sEH-H) and phosphatase (sEH-P) activities measured in renal tissue of WT and sEH-P KI rats. Mean and SEM values are shown and group effect was determined by Mann-Whitney test. ***P*<0.01.

**Figure S7.** Twelve-week survival after surgery of WT and sEH-P KI sham-operated and 5/6 nephrectomized rats (5/6Nx). Group effect was determined by log-rank test (Mantel-Cox). **P*<0.05 *vs.* Sham WT, ***P*<0.01 *vs.* Sham KI.

**Figure S8.** Urine volume (A) and water intake (F) measured 5 and 10 weeks after surgery in WT and sEH-P KI sham-operated and 5/6 nephrectomized rats (5/6Nx). Mean and SEM values are shown and group effect was determined by one-way *ANOVA* with Bonferroni's *post* *hoc* test. **P*<0.05, *****P*<0.0001.

**Figure S9.** Representative images of kidney Trichome Masson staining (A) and scoring of interstitial inflammation (B), tubular damage (C) and glomerular sclerosis (D) lesions in WT and sEH-P KI sham-operated and 5/6 nephrectomized rats (5/6Nx). Mean and SEM values are shown and group effect was determined by Kruskal-Wallis test with Dunn’s *post* *hoc* test. ***P*<0.01.

**Figure S10.** Plasma calcium-to-phosphorus product measured 5 and 10 weeks after surgery in WT and sEH-P KI sham-operated and 5/6 nephrectomized rats (5/6Nx). Mean and SEM values are shown and group effect was determined by one-way *ANOVA* with Bonferroni's *post* *hoc* test.
